# Hierarchical Nanosheets/Walls Structured Carbon‐Coated Porous Vanadium Nitride Anodes Enable Wide‐Voltage‐Window Aqueous Asymmetric Supercapacitors with High Energy Density

**DOI:** 10.1002/advs.201900550

**Published:** 2019-06-28

**Authors:** Jun Huang, Zhongyou Peng, Yingbo Xiao, Yazhou Xu, Lingfang Chen, Yushuai Xiong, Licheng Tan, Kai Yuan, Yiwang Chen

**Affiliations:** ^1^ Institute of Polymers and Energy Chemistry College of Chemistry Nanchang University 999 Xuefu Avenue Nanchang 330031 China

**Keywords:** aqueous asymmetric supercapacitors, energy density, nanosheet arrays, vanadium nitride, voltage window

## Abstract

The energy density of aqueous asymmetric supercapacitors (ASCs) is usually limited by low potential windows and capacitances of both anode and cathode. Herein, a facile strategy to fabricate hierarchical carbon‐coated porous vanadium nitride nanosheet arrays on vertically aligned carbon walls (CC/CW/p‐VN@C) as anode for aqueous ASCs is reported. The potential window of CC/CW/p‐VN@C electrode can be stably extended to –1.3 to 0 V (vs Ag/AgCl) with greatly improved specific capacitance (604.8 F g^−1^ at 1 A g^−1^), excellent rate capability (368 F g^−1^ at 60 A g^−1^), and remarkable electrochemical stability. To construct ASCs, a Birnessite Na_0.5_MnO_2_ nanosheet arrays (CC/CW/Na_0.5_MnO_2_) cathode is similarly built. Benefiting from the matchable potential windows and high specific capacitances of the rationally designed anode and cathode, aqueous CC/CW/p‐VN@C||CC/CW/Na_0.5_MnO_2_ ASCs with a wide voltage window of 2.6 V are fabricated. Moreover, the ASCs showcase an ultrahigh energy density up to 96.7 W h kg^−1^ at a high power density of 1294 W kg^−1^, and excellent cycling stability (92.5% retention after 10 000 cycles), outperforming most of previously reported ASCs and even comparable to that of organic electrolyte supercapacitors (SCs). This efficient strategy for fabricating 2.6 V aqueous ASCs suggests a promising research system for high energy density SCs.

## 1 Introduction

The current challenge of supercapacitors (SCs) is how to promote the energy density without sacrificing their high power density and outstanding cycle life.^1^ According to the key equation *E* = 1/2 *CV* ^2^, to achieve a theoretically high energy density (*E*), the operating voltage (*V*) and device capacitance (*C*) should be increased simultaneously.^2^ Constructing asymmetric supercapacitors (ASCs) with separated potential windows and high capacitances of anode and cathode is a promising strategy to boost the energy density. Numerous works have been devoted to the field of ASCs design, such as MnO_2_||graphene (G), MnO_2_||Fe_2_O_3_, polyaniline||WO_3‐x_@MoO_3‐x_, G‐RuO_2_||G, Co_3_O_4_||G, G‐Ni(OH)_2_||G, and NiCo_2_S_4_||G/carbon spheres,^3^, ^4^, ^5^, ^6^, ^7^, ^8^, ^9^ but the cell voltages of most previously reported ASCs are lower than 2.0 V. In addition, the power density is usually limited by using solid‐state electrolyte due to the sluggish kinetics.^10^, ^11^ Therefore, aqueous electrolyte with low cost, low toxicity, and high ionic conductivity remains unique advantages to high performance SCs.^12^, ^13^ However, the fundamental challenge of aqueous SCs is limited by the undesirable water decomposition at 1.23 V.^14^ To mitigate such limitation, neutral aqueous electrolyte such as Na_2_SO_4_ has recently shown to be effective for extending voltage window of aqueous SCs to 2.0 V.^15^, ^16^ Further boosting the working voltage of ASCs over 2.0 V still remains a great challenge due to the low hydrogen evolution reaction (HER)/oxygen evolution reaction (OER) overpotentials of anode/cathode. Thus, to construct a high‐voltage aqueous ASCs, it is essential to fabricate anode and cathode with respect to HER and OER, respectively.

The electrochemical performance of ASCs depends on the active materials of anode and cathode. Numerous works have reported that the pseudocapacitive materials with high specific capacitances were used as cathode, while carbon‐based materials with high surface area and remarkable electrical conductivity as anode.^17^, ^18^, ^19^ Among the pseudocapacitive cathode materials, MnO_2_ has been widely investigated because of its high theoretical specific capacitance, low cost, and high OER overpotential with a voltage window of 1.0 V.^20^ It has been proved that preinsertion of Na^+^ ion into MnO_2_ lattice can effectively increase the supercapacitive performance with large voltage window of 1.3 V and high specific capacitance of 366 F g^−1^ in the neutral electrolyte.^21^ Despite the great improvement in cathode, unfortunately, for anode, the low capacitance of carbon‐based materials do not match well with cathode, that eventually limit the performance of ASCs. Thus, it is urgently to explore alternative anode materials with suitable specific capacitance and high potential window to replace carbon‐based materials. Some metal oxides such as MoO_3_, Fe_2_O_3_, VO_2_, and SnO_2_ own higher specific capacitances than carbon‐based materials, and display similar potential windows with carbon‐based materials due to the sluggish kinetics of HER.^22^, ^23^, ^24^, ^25^ However, their poor electrical conductivities seriously limit the supercapacitive performance. Surprisingly, vanadium nitride (VN) with large potential window of –1.2 to 0 V, high theoretical specific capacitance of 1340 F g^−1^, and excellent electrical conductivity of 1.67 × 10^6^ Ω^−1^ m^−1^ exhibits as promising anode material for high‐performance ASCs.^26^, ^27^ Yet, during electrochemical reaction, the formation of vanadium oxide (VO_x_) on the surface of VN anode usually leads to poor electrochemical stability in KOH electrolyte.^28^ Besides, the low specific capacitances were achieved for previously reported VN‐based electrodes, which primarily blocked by the inefficient nanostructure.^26^, ^27^, ^28^, ^29^, ^30^ Up to now, the fabrication of VN‐based electrode with high specific capacitance and superb electrochemical stability still remains challenging. Thus, there are very few reports about VN‐based electrode as anode in high‐performance ASCs.

Herein, hierarchical carbon‐coated porous VN nanosheets on vertically aligned carbon walls (CC/CW/p‐VN@C) have been fabricated as anode for ASCs. The potential window of CC/CW/p‐VN@C electrode can be stably expanded to –1.3 to 0 V (vs Ag/AgCl) with greatly improved specific capacitance (605 F g^−1^ at 1 A g^−1^). Benefiting from the ultrathin carbon layer coating, the CC/CW/p‐VN@C electrode exhibits excellent rate capability (368 F g^−1^ at 60 A g^−1^) and remarkable cycling stability (90.5% retention after 10 000 cycles). To construct ASC, Birnessite Na_0.5_MnO_2_ nanosheet arrays electrode (CC/CW/Na_0.5_MnO_2_) was similarly built as cathode, which achieves a high specific capacitance (557 F g^−1^ at 1 A g^−1^) with a large potential window of 0−1.3 V (vs Ag/AgCl). Due to the matchable potential windows and high specific capacitances of both anode and cathode, a 2.6 V aqueous CC/CW/p‐VN@C||CC/CW/Na_0.5_MnO_2_ ASC has been fabricated with an ultrahigh energy density up to 96.7 W h kg^−1^ and excellent cycling stability (92.5% retention after 10 000 cycles), outperforming most of previously reported ASC devices and even comparable to that of organic electrolyte SCs.

## 2 Results and Discussion

### 2.1 Morphology and Structure Characterization

**Figure 1:**
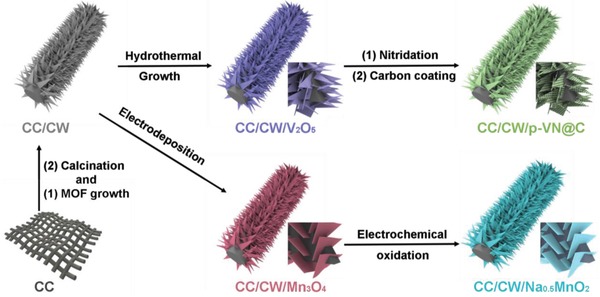
Schematic illustration of the synthesis procedure for hierarchical nanosheets/walls structured CC/CW/p‐VN@C anode and CC/CW/Na_0.5_MnO_2_ cathode.

**Figure 2:**
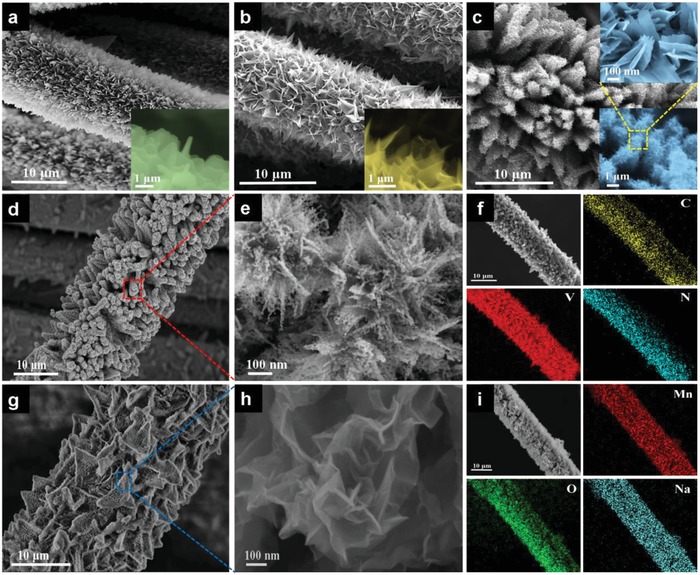
a–c) SEM images of CC/Co‐Zn MOF (a), CC/CW (b), and CC/CW/V_2_O_5_ (c) nanosheet arrays. d,e) SEM and magnified SEM images of CC/CW/p‐VN@C nanosheet arrays. f) EDS element mapping images of CC/CW/p‐VN@C nanosheet arrays. g,h) SEM and magnified SEM images of CC/CW/Na_0.5_MnO_2_ nanosheet arrays. i) EDS element mapping images of CC/CW/Na_0.5_MnO_2_ nanosheet arrays.

The schematic fabrication processes of hierarchical nanosheets/walls structured CC/CW/p‐VN@C and CC/CW/Na_0.5_MnO_2_ electrodes are shown in **Figure** **1**. The commercial woven carbon cloth (CC) was used as conductive substrate. The carbon wall arrays were growth on the surface of CC (CC/CW), which severed as secondary substrate for further growth of carbon‐coated porous VN and Na_0.5_MnO_2_ nanosheet arrays (denoted as CC/CW/p‐VN@C and CC/CW/Na_0.5_MnO_2_, respectively), detailed synthesis procedures are given in the Experimental Section. Scanning electron microscopy (SEM) images reveal that the vertically aligned Co‐Zn metal‐organic framework (MOF) walls were uniformly wrapped on the entire surface of CC with densely packed and highly ordered morphology (**Figure** **2**a). After annealing, the Co‐Zn MOF walls were converted into vertically aligned carbon walls with smooth surface, and with ≈100 nm in thickness and about 2.5 µm in lateral size (Figure 2b). The low‐magnification SEM images of CC/CW reveal that the carbon wall arrays can be uniformly prepared on CC in a large scale (Figure S1, Supporting Information). As shown in Figure 2c, after growth of V_2_O_5_, the surface of carbon walls was decorated by interconnected V_2_O_5_ thin nanosheets with lateral size of about 400 nm, which form a hierarchical nanosheets/walls structure. Besides, the nanosheets/walls structure can still be well retained even in a large scale (Figure S2, Supporting Information). Furthermore, the nanosheets/walls structure was well preserved after nitridation while the smooth surface of nanosheets became a rough and porous morphology (Figure S3, Supporting Information). After treated with glucose solution, the porous nanosheets were coated with a uniform and continuous film (Figure S4, Supporting Information). Finally, CC/CW/p‐VN@C was obtained after further calcination. The carbon wall arrays and the interconnected porous nanosheet structure were retained without obvious destruction (Figure 2d,e). For comparison, CC/p‐VN was also prepared without carbon walls on CC as a secondary substrate. Porous VN nanowires were uniformly coated on CC with much lower mass loading of VN than that of CC/CW/p‐VN@C (Figure S5, Supporting Information). The energy dispersive X‐ray spectroscopy (EDS) element mapping images exhibit a homogeneous distribution of the C, V, and N elements throughout CC/CW/p‐VN@C (Figure 2f).

The CC/CW/Na_0.5_MnO_2_ electrode was similarly built with carbon walls as secondary substrate. The ultrafine cross‐linked Mn_3_O_4_ nanosheets with smooth surface were uniformly deposited on the carbon walls (Figure S6, Supporting Information). Similarly, in the absence of carbon walls, the low mass loading of Mn_3_O_4_ nanosheets was coated on CC (Figure S7, Supporting Information). After electrochemical oxidation of Mn_3_O_4_ nanosheets in Na_2_SO_4_ electrolyte, the Na_0.5_MnO_2_ nanosheets were in situ formed with highly wrinkled heterostructures (Figure 2g,h), which is beneficial for exposing much more active sites for fast faradaic reactions. The corresponding EDS mapping images reveal that all the elements are homogenously distributed along the whole CC/CW/Na_0.5_MnO_2_ (Figure 2i), suggesting that Na^+^ is uniformly intercalated into Mn_3_O_4_ during the electrochemical oxidation.

**Figure 3:**
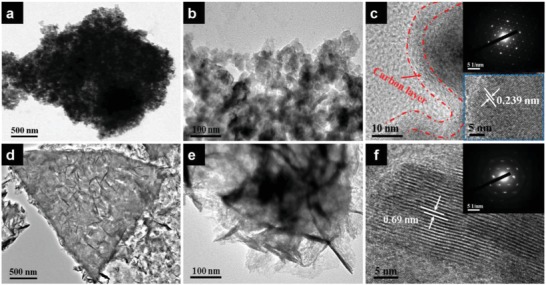
a,b) TEM images of CW/p‐VN@C (a) and porous p‐VN@C (b) nanosheets. c) Magnified TEM image of p‐VN@C nanosheet, and the insets show the corresponding SAED pattern (upper right) and HRTEM image (bottom right). d,e) TEM images of CW/Na_0.5_MnO_2_ (d) and Na_0.5_MnO_2_ (e) nanosheets. f) HRTEM image of Na_0.5_MnO_2_ nanosheet, and the inset shows the corresponding SAED pattern.

The microstructures of CC/CW/p‐VN@C and CC/CW/Na_0.5_MnO_2_ nanosheet arrays were further measured by transmission electron microscopy (TEM). **Figure** **3**a presents the TEM image of a single CW/p‐VN@C, displaying a triangle shape with a lateral size of 2–3 µm, which matches well with the size of carbon wall. The nanosheet is assembled by numerous interconnected VN nanocrystallines with an average size of about 20 nm (Figure 3b). Besides, there are numerous pores between these nanocrystallines, indicating a porous structure of VN nanosheet. Such porous structure provide high open specific surface for contacting with electrolyte, thus enhance fast transfer of ions and electrons. The high‐resolution TEM (HRTEM) reveals that the VN nanosheets were covered by a continuous amorphous carbon shell, with an average thickness of about 5 nm (Figure 3c). The HRTEM image reveals a lattice fringe of 0.239 nm, which is consistent with the d‐spacing of (111) plane of the cubic VN structure. The selected area electron diffraction (SAED) pattern (inset in Figure 3c) indicates a single crystalline feature and can be well indexed to the cubic VN.

In order to understand the phase and chemical compositional evolution of the samples upon thermal reduction, the corresponding X‐ray diffraction (XRD) patterns of CC/CW, CC/CW/V_2_O_5_, and CC/CW/p‐VN@C were investigated (Figure S8, Supporting Information). The characteristic peaks appear at 26° and 44°, which are attributed to the MOF‐derived carbon walls and CC substrate.^31^ The positions of diffraction peaks in the XRD pattern for CC/CW/p‐VN@C are close to those of cubic VN (JCPDS card No. 73–0528), confirming V_2_O_5_ was successfully transformed into cubic VN.^27^ The broaden diffraction peaks suggest the small size of individual VN crystallite. The chemical compositions and valence states of the CC/CW/p‐VN@C were analyzed by X‐ray photoelectron spectroscopy (XPS; Figure S9, Supporting Information). The full XPS spectrum contains the signals of C, V, and N, displaying their coexistence in the CC/CW/p‐VN@C. The high‐resolution V 2p XPS spectra show three chemical states of V. The peaks at 513.7 and 521.1 eV correspond to V–N, the peaks at 514.8 and 521.8 eV correspond to V–N–O, and the peaks at 516.6 and 523.9 eV correspond to V–O. The high‐resolution N1s spectra show two different states. The peaks at 397.2 and 399.1 eV are attributed to N from vanadium oxynitride and VN, respectively.^26^

For CC/CW/Na_0.5_MnO_2_ nanosheet arrays, as shown in Figure 3d, obviously, the ultrathin wrinkled Na_0.5_MnO_2_ nanosheets were uniformly anchored on the surface of CW. The Na_0.5_MnO_2_ nanosheets are 5−10 nm in thickness and 100−200 nm in lateral size (Figure 3e). The HRTEM image indicates well‐resolved lattice fringes with an interplanar spacing of 0.69 nm (Figure 3f), which is well agree with (001) plane (≈0.7 nm) of Birnessite MnO_2_.^32^ The large interlayer distance reveals that crystal water exists between the MnO_6_ octahedral layers.^33^ The layered Birnessite structure of the Na_0.5_MnO_2_ was further confirmed by the SAED pattern of the nanosheet (inset of Figure 3f). From the XRD patterns of the CC/CW/Mn_3_O_4_ and in situ formed CC/CW/Na_0.5_MnO_2_ after electrochemical oxidation (Figure S10, Supporting Information), all diffraction peaks of Mn_3_O_4_ can be well indexed to the tetrahedral hausmannite Mn_3_O_4_. After electrochemical oxidation, the newly emerged diffraction peaks can be indexed to monoclinic Birnessite. These results were further confirmed by XPS analysis (Figure S11, Supporting Information), which are consistent with previously reported results.^34^

### 2.2 Electrochemical Properties

**Figure 4:**
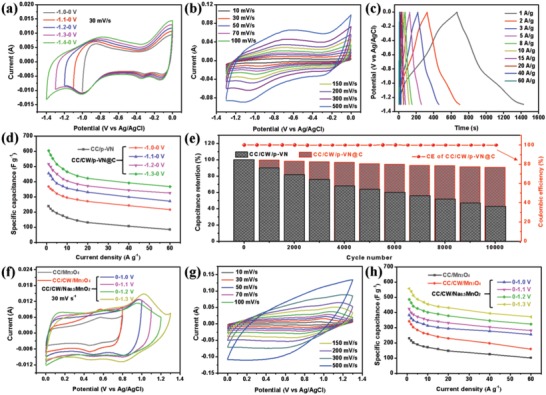
a) The CV curves of CC/CW/p‐VN@C anode in different potential windows of –1.0 to 0, –1.1 to 0, –1.2 to 0, –1.3 to 0, and –1.4 to 0 V at a same scan rate of 30 mV s^−1^. b) CV curves of CC/CW/p‐VN@C anode between –1.3 and 0 V at different scan rates. c) The GCD curves of CC/CW/p‐VN@C anode in –1.3 to 0 V at different current densities. d) The specific capacitances of CC/p‐VN in –1.3 to 0 V, and CC/CW/p‐VN@C electrodes in different potential windows of –1.0 to 0, –1.1 to 0, –1.2 to 0, and –1.3 to 0 V at different current densities. e) Cycle performances of CC/CW/p‐VN and CC/CW/p‐VN@C electrodes in –1.3 to 0 V and the coulombic efficiency of CC/CW/p‐VN@C electrode. f) The CV curves of CC/Mn_3_O_4_, CC/CW/Mn_3_O_4_ in a potential window of 0‒0.8 V and CC/CW/Na_0.5_MnO_2_ electrode in different potential windows of 0‒1.0, 0‒1.1, 0‒1.2, and 0‒1.3 V at a same scan rate of 30 mV s^−1^. g) CV curves of CC/CW/Na_0.5_MnO_2_ cathode between 0 and 1.3 V at different scan rates. h) The specific capacitances of CC/Mn_3_O_4_, CC/CW/Mn_3_O_4_ in a potential window of 0‒0.8 V and CC/CW/Na_0.5_MnO_2_ electrode in different potential windows of 0‒1.0, 0‒1.1, 0‒1.2, and 0‒1.3 V at different current densities.

The electrochemical performance of the hierarchical CC/CW/p‐VN@C anode and CC/CW/Na_0.5_MnO_2_ cathode was first measured in a three‐electrode system in 1 m Na_2_SO_4_ electrolyte. As shown in **Figure** **4**a, typical cyclic voltammetry (CV) curves of CC/CW/p‐VN@C were recorded in various voltage windows of –1.0 to 0, –1.1 to 0, –1.2 to 0, –1.3 to 0, and –1.4 to 0 V at a scan rate of 30 mV s^−1^. Obviously, it was found that the potential window of the CC/CW/p‐VN@C electrode can be stably extended to –1.3 to 0 V (vs Ag/AgCl), providing the possibility to construct high voltage window of aqueous ASCs. Besides, the CV curves of CC, CC/CW, and CC/CW/p‐VN@C electrodes were compared to distinguish the capacitance contribution of substrates (Figure S12, Supporting Information), demonstrating that the capacitance of CC/CW/p‐VN@C electrode mainly owes to the faradaic reaction of VN (the capacitance of CC and CC/CW in CC/CW/p‐VN@C electrode is about 2.1% and 8.6%, respectively). To further investigate the stable potential window under –1.3 to 0 V, the CV test at various scan rates was carried out (Figure 4b). Even at a high scan rate of 500 mV s^−1^, the shape of CV curves retained as well, indicating the superb reversibility and rate capability of the CC/CW/p‐VN@C electrode. This result was further confirmed by cycle performance recorded by the CV curves (Figure S13, Supporting Information). Besides, the symmetric galvanostatic charge/discharge (GCD) curves of the CC/CW/p‐VN@C anode at different current densities are arranged from 1 to 60 A g^−1^, further confirming the high rate performance (Figure 4c).

In order to prove the superior capacitive performance of hierarchical carbon‐coated porous CC/CW/p‐VN@C anode, the electrochemical performance of CC/p‐VN (Figure S14, Supporting Information) and CC/CW/p‐VN@C (Figure S15, Supporting Information) electrodes in various voltage windows was also measured at different scan rates (from 10 to 500 mV s^−1^) and current densities (from 1 to 60 A g^−1^). The specific capacitance of CC/p‐VN and CC/CW/p‐VN@C in various voltage windows is compared in Figure 4d. At a current density of 1 A g^−1^, the specific capacitance of the CC/CW/p‐VN@C electrode in –1.3 to 0 V can reach 605 F g^−1^, which is higher than 517, 455, and 368 F g^−1^ in –1.2 to 0, –1.1 to 0, and –1.0 to 0 V, respectively. The specific capacitance of CC/CW/p‐VN@C is also much higher than that of 239 F g^−1^ for CC/p‐VN electrode. Besides, even at a high current density of 60 A g^−1^, the CC/CW/p‐VN@C anode exhibits eminent rate performance under –1.3 to 0 V with a high specific capacitance of 367 F g^−1^, which is much larger than CC/p‐VN electrode (85 F g^−1^ at 60 A g^−1^) and those of previously reported VN‐based electrodes.^26^, ^27^, ^28^, ^29^, ^30^, ^35^, ^36^, ^37^, ^38^, ^39^ To investigate the function of carbon layer coating, the long‐term cycling performances of the CC/CW/p‐VN@C and CC/CW/p‐VN electrodes were carried out for 10 000 cycles at a current density of 5 A g^−1^ (Figure 4e). After 10 000 cycles, the CC/CW/p‐VN@C electrode still can retain 90.5% of its initial capacitance and nearly 100% coulombic efficiency, while only 43.2% of initial capacitance maintained for CC/CW/p‐VN electrode, indicating an excellent electrochemical stability can be achieved by the thin carbon layer coating. This was also demonstrated by the electrochemical impedance spectroscopy (EIS) and CV measurements of CC/CW/p‐VN and CC/CW/p‐VN@C electrodes before and after 10 000 cycles (Figure S16, Supporting Information). Besides, the morphology of CC/CW/p‐VN@C was conserved well without obvious desquamation after 10 000 cycles (Figure S17, Supporting Information). XPS analyses indicated that the VN in CC/CW/p‐VN electrode was oxidized to VO_x_ after cycling measurement (Figure S18a, Supporting Information). Core level V 2p and N 1s spectra showed that no obvious compositional modification of VN in CC/CW/p‐VN@C electrode was found after cycling measurement (Figure S18, Supporting Information). These results confirmed that the thin carbon layer coating can effectively suppress the irreversible oxidation reaction and structural pulverization of VN without sacrificing their electrochemical performances.

To further investigate the electrochemical stability of the CC/CW/p‐VN@C electrode, long‐term cycling performance was also investigated under various voltage windows for 10 000 cycles at a current density of 5 A g^−1^ (Figure S19, Supporting Information). Negligible capacitance fading was found in different potential windows, indicating excellent electrochemical stability of the CC/CW/p‐VN@C electrode. The hierarchical nanosheets/walls structure and the carbon‐coated porous architecture can be responsible for the superior electrochemical performance of CC/CW/p‐VN@C anode. These aforementioned results demonstrating the remarkable capacitive performance of the uniquely engineered CC/CW/p‐VN@C anode with a large potential window of –1.3 to 0 V is promising for developing large voltage window and high energy density aqueous ASCs.

Similarly, the electrochemical performance of the nanosheets/walls structured CC/CW/Na_0.5_MnO_2_ cathode was also investigated in a three‐electrode cell with 1 m Na_2_SO_4_ electrolyte in various potential window of 0–1.0, 0–1.1, 0–1.2, and 0–1.3 V (vs Ag/AgCl). To conform the superb electrochemical performance of CC/CW/Na_0.5_MnO_2_ cathode, the CC/Mn_3_O_4_ and CC/CW/Mn_3_O_4_ electrodes were first compared by CV and GCD measurements (Figure S20, Supporting Information). The carbon walls serve as secondary substrate that can greatly increase the mass loading and provide more active sites for energy storage. Besides, the capacitance of CC and CC/CW in CC/CW/Na_0.5_MnO_2_ electrode is about 1.4% and 7.3%, respectively, demonstrating that the high capacitance of CC/CW/Na_0.5_MnO_2_ electrode mainly owes to the faradaic reaction of Na_0.5_MnO_2_ (Figure S21, Supporting Information). The comparison of CV curves for CC/CW/Na_0.5_MnO_2_, CC/CW/Mn_3_O_4_, and CC/Mn_3_O_4_ are displayed in Figure 4f, showing the largest potential window and highest specific capacitance of the hierarchical nanosheets/walls structured CC/CW/Na_0.5_MnO_2_ cathode. After electrochemical oxidation of CC/CW/Mn_3_O_4_, the potential window of CC/CW/Na_0.5_MnO_2_ electrode can efficiently be extended from 0–0.8 to 0–1.3 V. The quasirectangular shape of CV curves of the CC/CW/Na_0.5_MnO_2_ electrode was well preserved at different scan rates from 10 to 500 mV s^−1^ (Figure 4g), revealing excellent rate capability and fast charge‐transfer kinetics, which was further confirmed by the GCD measurements (Figure S22, Supporting Information).

To get insight to the rate performance of the CC/CW/Na_0.5_MnO_2_ cathode in various voltage windows, CV measurements at different scan rates from 10 to 500 mV s^−1^ and GCD measurements at various current densities from 1 to 60 A g^−1^ were carried out (Figure S23, Supporting Information). The CV and GCD curves of the CC/CW/Na_0.5_MnO_2_ cathode retain ideal rectangular and symmetric shape even at a high scan rate of 300 mV s^−1^ and a high current density of 60 A g^−1^, respectively, indicating excellent rate performance and high reversibility. To confirm the improved capacitive performance of the hierarchical nanosheets/walls structured CC/CW/Na_0.5_MnO_2_ cathode, the capacitances for CC/Mn_3_O_4_ and CC/CW/Mn_3_O_4_ in a potential window of 0‒0.8 V and CC/CW/Na_0.5_MnO_2_ electrode in various potential windows of 0‒1.0, 0‒1.1, 0‒1.2, and 0‒1.3 V were compared in Figure 4h. At a current density of 1 A g^−1^, the specific capacitance of the CC/CW/Na_0.5_MnO_2_ electrode can reach 557 F g^−1^ in 0‒1.3 V, which is higher than 486, 425, and 383 F g^−1^ in 0‒1.2, 0‒1.1, and 0‒1.0 V, respectively, also much higher than CC/CW/Mn_3_O_4_ electrode with 342 F g^−1^ and CC/Mn_3_O_4_ electrode with 230 F g^−1^. Especially, even at a high current density of 60 A g^−1^, the CC/CW/Na_0.5_MnO_2_ nanosheet electrode can still retain a large specific capacitance of 371 F g^−1^, displaying excellent rate performance under 0‒1.3 V. The value of the capacitance is larger than those of previously reported MnO_2_‐based electrodes at similar current densities.^4^, ^16^, ^21^, ^31^, ^34^, ^40^, ^41^, ^42^, ^43^ In addition, the long‐term cycling test at a current density of 5 A g^−1^ was carried out, and 94.5% of the initial capacitance remained after 10 000 cycles (Figure S24, Supporting Information), indicating the excellent cycling stability of the CC/CW/Na_0.5_MnO_2_ cathode. Thus, the suitable system with appropriate potential windows and high capacitances of both CC/CW/p‐VN@C anode and CC/CW/Na_0.5_MnO_2_ cathode is fit for construct highly efficient aqueous ASCs.

**Figure 5:**
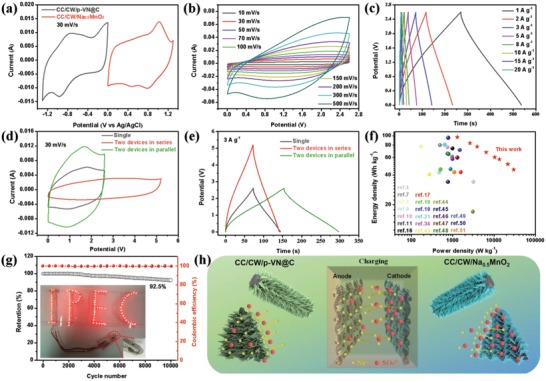
a) CV curves of CC/CW/p‐VN@C anode and CC/CW/Na_0.5_MnO_2_ cathode in separate potential windows at a same scan rate of 30 mV s^−1^. b) CV curves of as‐assembled aqueous ASC at different scan rates from 10 to 500 mV s^−1^. c) GCD curves of the ASC between 0 and 2.6 V at different current densities. d) CV and e) GCD curves of a single ASC and two ASCs in series or parallel, respectively. f) Ragone plot related to energy and power densities of the ASC compared with literature results. g) Cycle performance and coulombic efficiency of the ASC device. The inset photograph exhibits a series of LED arrays in parallel powered up by a single ASC cell. h) The electrode design and charging process of the CC/CW/p‐VN@C anode and the CC/CW/Na_0.5_MnO_2_ cathode in the present aqueous ASC system.

To confirm suitable operation of the hierarchical nanosheets/walls structured electrodes in fully functional devices, aqueous ASCs were assembled by CC/CW/p‐VN@C anode and CC/CW/Na_0.5_MnO_2_ cathode with 1 m Na_2_SO_4_ as electrolyte. Given that the CC/CW/p‐VN@C anode and CC/CW/Na_0.5_MnO_2_ cathode possess stable potential windows of –1.3 to 0 V and 0−1.3 V, respectively, a theoretical 2.6 V ASC can be achieved (**Figure** **5**a). As expected, the CV curves with different potential window from 1.0 to 2.6 V at 30 mV s^−1^ (Figure S25a, Supporting Information) and GCD curves with different voltage windows at 1 A g^−1^ (Figure S25b, Supporting Information) of the as‐fabricated ASC reveal that the device can stably and effectively work at 2.6 V. No obvious H_2_/O_2_ evolution in the CV plots and no overcharging region in the GCD plots were observed, implying the excellent electrochemical operation stability at wide potential window of 2.6 V. Besides, the CV curves at various scan rates from 10 to 500 mV s^−1^ maintain the rectangular shape without severe distortion, demonstrating the superb rate performance and fast charge/discharge ability of the ASC (Figure 5b). The EIS spectrum of the ASC shows a small charge transfer resistance of 0.31 Ω (Figure S26, Supporting Information), suggesting fast Faradaic reactions in both cathode and anode.

To further evaluate the electrochemical performance of the ASC device, GCD measurements at various current densities from 0 to 2.6 V were also recorded (Figure 5c). The specific capacitance of the ASC can reach 103 F g^−1^ at 1 A g^−1^ between 0 and 2.6 V (Figure S27, Supporting Information). To demonstrate the integration of our manufacturing strategy, two ASCs in series and parallel were also measured. As shown in Figure 5d,e, two ASCs in series exhibited a 5.2 V voltage window with identical discharge time for a single ASC device, and those in parallel exhibited an increased capacitance of two times of a single device. These results roughly obey the basic rule of series and parallel connections of capacitors, indicating the excellent performance uniformity of our ASCs. Benefiting from high specific capacitance and large cell voltage, the present ASC can deliver a maximum energy density of 96.7 W h kg^−1^ at a high power density of 1294 W kg^−1^ (Figure 5f), which is higher than the values of most previously reported ASCs.^4^, ^7^, ^8^, ^9^, ^10^, ^11^, ^16^, ^17^, ^18^, ^19^, ^21^, ^34^, ^43^, ^44^, ^45^, ^46^, ^47^, ^48^, ^49^, ^50^, ^51^ To further investigate the stable working voltage window of 0–2.6 V, the long‐term charge/discharge cycling test was carried out for 10 000 cycles at 10 A g^−1^. As shown in Figure 5g, an excellent capacitance retention of 92.5% was achieved, with near 100% coulombic efficiency after 10 000 cycles, indicating the stable operation within the suggested potential window and the prominent energy storage capability of the ASC device. Benefiting from the high energy density, a single ASC device can efficient light a series of LED arrays in parallel (inset of Figure 5g).

To investigate the potential wearable application of our high voltage ASCs, we fabricated the all‐solid‐state ASCs based on carboxymethyl cellulose sodium (CMC)‐Na_2_SO_4_ gel electrolyte (Figure S28, Supporting Information). Due to the relatively low electrical conductivity of CMC‐Na_2_SO_4_ gel electrolyte compared with aqueous Na_2_SO_4_ electrolyte, the working voltage of all‐solid‐state ASC only can reach to 2.4 V. The specific capacitance of the all‐solid‐state ASC is 75 F g^−1^ at 1 A g^−1^ and exhibits a maximum energy density of 60 W h kg^−1^ at a power density of 1200 W kg^−1^. Furthermore, a series of mechanical flexibility tests were also performed, as illustrated in Figure S28f, Supporting Information; negligible performance degradation and almost completely overlapped CV curves were achieved at a scan rate of 30 mV s^−1^ with different bending conditions, highlighting the exceptional flexibility and stability of our all‐solid‐state ASCs.

The rational electrode designs and cooperation of both anode and cathode are responsible for the remarkable capacitive performance of the as‐fabricated aqueous ASC device with 2.6 V working voltage, which are schematically illustrated in Figure 5h. The smart structure engineering of both anode and cathode endows the ASC remarkable electrochemical performance, which can be attributed to the following advantages. First, the carbon wall arrays served as secondary substrate for growing active materials with higher mass loading, which is simultaneously efficient for both anode and cathode, resulting in higher specific capacitances. Second, for anode, the porous nature of VN nanosheet arrays not only can provide high specific surface but also can maximally expose active sites for efficient faradaic reaction, resulting in high specific capacitance, and shorten diffusion path of ion/electron for high power density. In addition, the thin carbon coating layer reinforce the electrochemical stability of anode. Third, in terms of cathode, the in situ formed Brinessite Na_0.5_MnO_2_ owns large layer spacing, facilitating fast Faradic reaction, assisting with the highly wrinkled nanosheets structure, resulting in high potential window and large capacitance. Furthermore, the reasonable match of anode and cathode with both appropriate potential windows and specific capacitances can achieve the theoretically highest voltage based on the separated potential windows of anode and cathode, and maximize the energy and power density.

## 3 Conclusion

In summary, hierarchical nanosheets/walls structured carbon‐coated porous VN nanosheets growth on vertically aligned carbon walls (CC/CW/p‐VN@C) were fabricated as anode for wide‐potential‐window aqueous ASCs with high energy density. Benefiting from the unique structure and morphology, highly utilization rate of porous VN nanosheets and the thin carbon layer coating, the as‐fabricated CC/CW/p‐VN@C anode exhibits a wide voltage window of –1.3 to 0 V with significantly improved specific capacitance (604.8 F g^−1^ at 1 A g^−1^), excellent rate capability (368 F g^−1^ at 60 A g^−1^), and remarkable cycling stability. The rational electrode design has also been successfully applied to prepare CC/CW/Na_0.5_MnO_2_ cathode with remarkable capacitive performance. Thus, thanks to the well‐separated potential windows and matchable specific capacitances, a 2.6 V aqueous CC/CW/p‐VN@C||CC/CW/Na_0.5_MnO_2_ ASC has been achieved. The ASC displays a high energy density up to 96.7 W h kg^−1^ and excellent cycling stability (92.5% retention after 10 000 cycles). Such a high working voltage and enhanced energy density is much higher than most of previously reported ASC devices and even comparable to that of organic electrolyte SCs. Given the synergistic effect of perfectly matched anode and cathode, high‐voltage aqueous ASCs with high energy density can be developed for various energy‐related applications.

## 4 Experimental Section

*Synthesis of CC/CW*: Typically, 40 mL of aqueous solution containing 2 mm of Co(NO_3_)_2_·6H_2_O and 1 mm of Zn(NO_3_)_2_·6H_2_O was mixed with 40 mL of aqueous solution containing 20 mm of 2‐methylimidazole under magnetic stirring. Then, a piece of CC (2 cm × 5 cm) was placed into the mixture and kept for 4 h at room temperature, followed by washing with DI water and dried in oven at 60 °C to product CC/Co‐Zn MOF. Finally, the CC/Co‐Zn MOF was annealed in air at 350 °C for 2 h to obtain CC/CW.

*Synthesis of CC/CW/p‐VN@C*: A total of 1.5 mm of V_2_O_5_ and 5 mm of H_2_C_2_O_4_ were dissolved in 10 mL of DI water at 75 °C. After the solution become dark blue, 2 mL of 30% H_2_O_2_ and 40 mL of ethanol were added and continuously stirred for 30 min, and then transferred to a 100 mL Teflon‐lined autoclave. A piece of CC/CW was immersed into the precursor solution in the autoclave. The autoclave was heated at 180 °C for 5 h, and cool down to room temperature. The obtained sample was washed with DI water and dried in oven at 60 °C, denoted as CC/CW/V_2_O_5_. Porous CC/CW/p‐VN nanosheets were obtained by annealing the as‐prepared CC/CW/V_2_O_5_ in NH_3_ at 600 °C for 1 h. The CC/p‐VN was synthesized with the same process. The CC/CW/p‐VN@C was synthesized by immersing the CC/CW/p‐VN into a 0.04 m glucose aqueous solution for 10 h with subsequent calcination in N_2_ atmosphere at 500 °C for 2 h. The mass loading of p‐VN@C is about 1.4 mg cm^−2^.

*Synthesis of CC/CW/Na_0.5_MnO_2_*: The Na_0.5_MnO_2_ nanosheets were prepared on CC/CW by a electrodeposition process in a three‐electrode system according to literature procedure.^21^ The free‐standing CC/CW (2 cm × 2 cm) was used as a working electrode, a platinum foil and an Ag/AgCl electrode was used as a counter electrode and reference electrode, respectively. A solution of 0.1 m Mn(CH_3_COO)_2_ and 0.1 m Na_2_SO_4_ was used as the electrolyte. The electrodeposition was carried out at a potential window of –1.8 to 0 V at 5 mV s^−1^ for two cycles at room temperature, and aged for 2 h in air to obtain CC/CW/Mn_3_O_4_. The CC/Mn_3_O_4_ was synthesized with the same process. The final CC/CW/Na_0.5_MnO_2_ was prepared by electrochemical oxidation between 0 and 1.3 V (vs Ag/AgCl) in 1 m Na_2_SO_4_ electrolyte for 500 cycles. The mass loading of Na_0.5_MnO_2_ is about 1.2 mg cm^−2^.

*Materials Characterization*: A field‐emission scanning electron microscope (FEI, QuanTA‐200F), TEM (JEOL, JEM‐2100F), and HRTEM were used to investigate the morphology and microstructure of these samples. The XRD (PERSEE, XD‐3 with Cu Kα radiation), EDS (Tecnai G2 F30 S‐TWIN), and XPS (Thermo‐VG; ESCALAB 250) were used to investigate crystallographic information and phase purity of these samples.

*Electrochemical Measurements*: The electrochemical characterizations were evaluated by CV, GCD, and EIS measurements using a CHI 660D electrochemical workstation (Chenhua, Shanghai) in a three‐electrode configuration for anode and cathode, and in a two‐electrode configuration for asymmetric SC devices, respectively. The asymmetric SC devices were fabricated with the CC/CW/p‐VN@C as anode and CC/CW/Na_0.5_MnO_2_ as cathode, respectively. A porous polymer membrane was used as the separator (Celgard 3501), and 1 m Na_2_SO_4_ solution was used as the electrolyte for aqueous devices and carboxymethyl cellulose sodium (CMC)‐Na_2_SO_4_ gel electrolyte for all‐solid‐state devices. The CMC‐Na_2_SO_4_ gel electrolyte was prepared by adding 10 g of Na_2_SO_4_ and 6 g of CMC into 100 mL of distilled water under stirring at 85 °C for 3 h until the gel became transparent.

## Conflict of Interest

The authors declare no conflict of interest.

## Supporting information

Supplementary
