# Supplementary material for: Hierarchical Nanosheets/Walls Structured Carbon‐Coated Porous Vanadium Nitride Anodes Enable Wide‐Voltage‐Window Aqueous Asymmetric Supercapacitors with High Energy Density
Source: Adv Sci (Weinh). 2019 Jun 28;6(16):1900550. doi: 10.1002/advs.201900550 (PMC6702630; doi:10.1002/advs.201900550)
Supplement: Supplementary file 1 — Supplementary [file ADVS-6-1900550-s001.pdf]

Copyright WILEY-VCH Verlag GmbH & Co. KGaA, 69469 Weinheim, Germany, 2019.

ADVANCED SCIENCE
Open Access

Supporting Information

for *Adv. Sci.,* DOI: 10.1002/advs.201900550

Hierarchical Nanosheets/Walls Structured Carbon-Coated Porous Vanadium Nitride Anodes Enable Wide-Voltage-Window Aqueous Asymmetric Supercapacitors with High Energy Density

Jun Huang, Zhongyou Peng, Yingbo Xiao, Yazhou Xu, Lingfang Chen, Yushuai Xiong, Licheng Tan, Kai Yuan, and Yiwang Chen**

Copyright WILEY-VCH Verlag GmbH & Co. KGaA, 69469 Weinheim, Germany, 2019.

Supporting Information

Hierarchical Nanosheets/Walls Structured Carbon Coated Porous Vanadium Nitride Anodes Enable Wide-Voltage-Window Aqueous Asymmetric Supercapacitors with High Energy Density

Jun Huang, Zhongyou Peng, Yingbo Xiao, Yazhou Xu, Lingfang Chen, Yushuai Xiong, Licheng Tan, Kai Yuan, and Yiwang Chen**

***Calculations*:**

Prior to the fabrication of the asymmetric supercapacitor, the mass loading of the cathode and anode were balanced according to the following equation:

$$\frac{m_+}{m_-} = \frac{C_{S-}\Delta V_-}{C_{S+}\Delta V_+} \qquad (1)$$

where *m* is the mass, *Cs* is the specific capacitance, and *ΔV* is the voltage range for positive and negative electrodes, respectively.

The specific capacitance (*C*) was calculated by the following equation:

$$C = \frac{I}{mdV/dt} \qquad (2)$$

where *I* (A) is the discharge current, *m* (g) represents the mass of the active material, and the value of *dV* (V)/*dt* (s) indicates the slope of the discharge curve in the GCD measurement.

Based on the total mass of the active materials of the anode and cathode, the energy density (*E*) and power density (*P*) were calculated using the following equations, respectively:

$$E = \frac{C\ (\Delta V)^2}{2 \times 3.6} \tag{3}$$

$$P = \frac{E \times 3600}{\Delta t} \tag{4}$$

where C (F g^{-1}) is the specific capacitance, ΔV (V) is the potential window, and Δt (s) is the discharge time. The units of energy density (E) and power density (P) are W h kg^{-1} and W kg^{-1}, respectively.

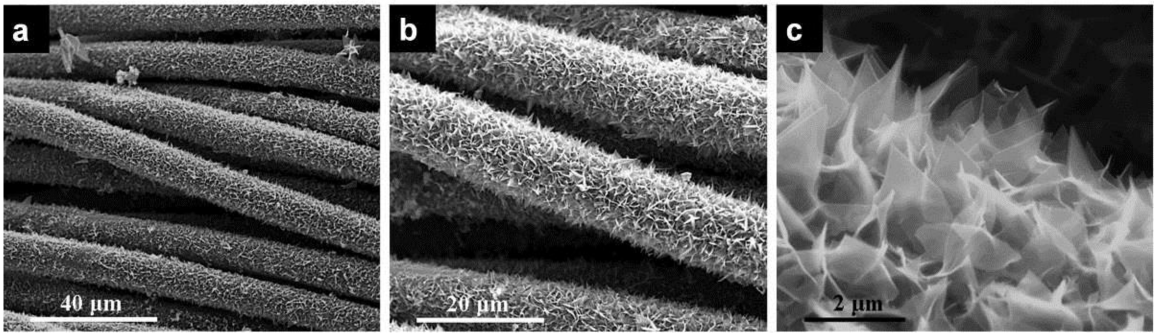

Figure S1. SEM images of CC/CW.

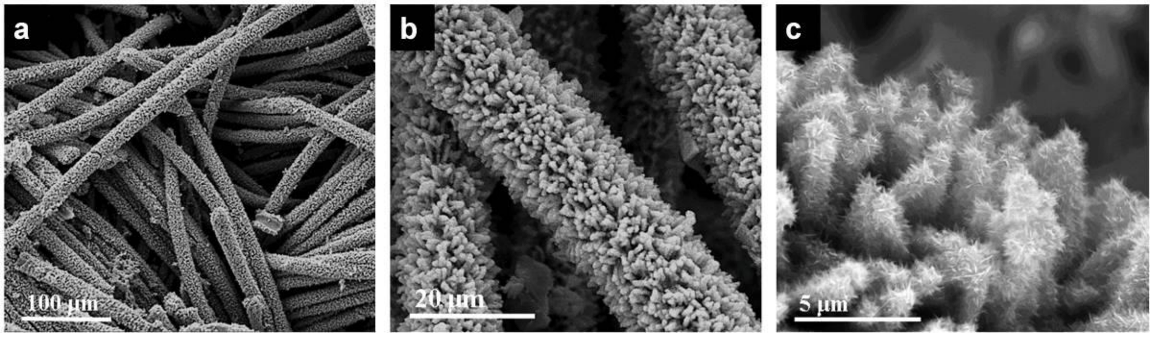

Figure S2. SEM images of $CC/CW/V_2O_5$.

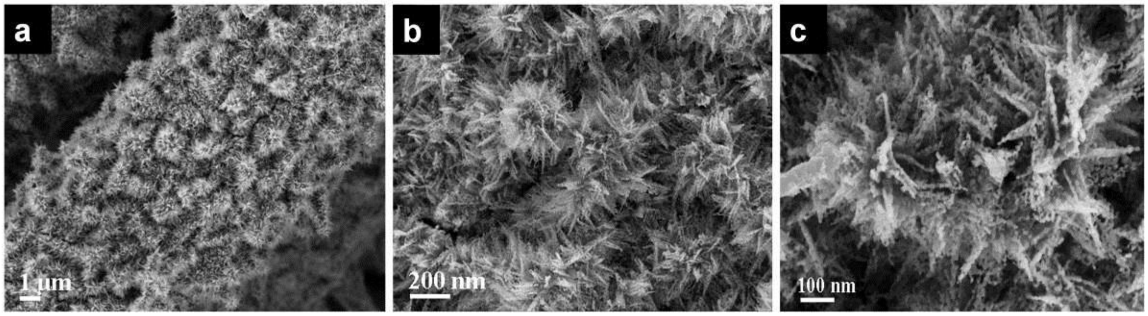

Figure S3. SEM images of CC/CW/p-VN.

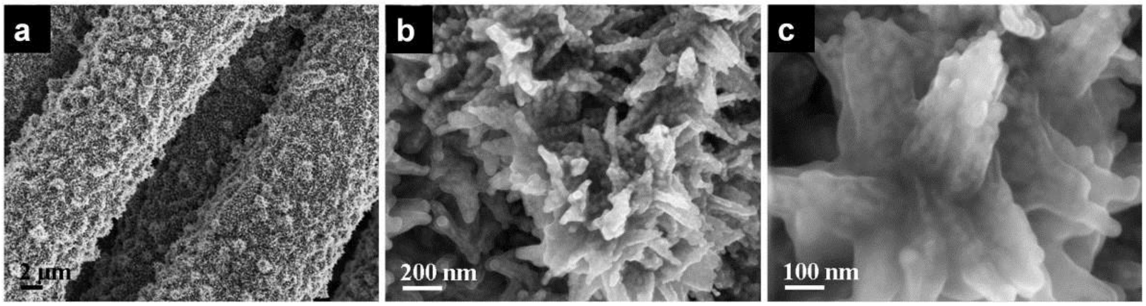

Figure S4. SEM images of CC/CW/p-VN@C without calcination.

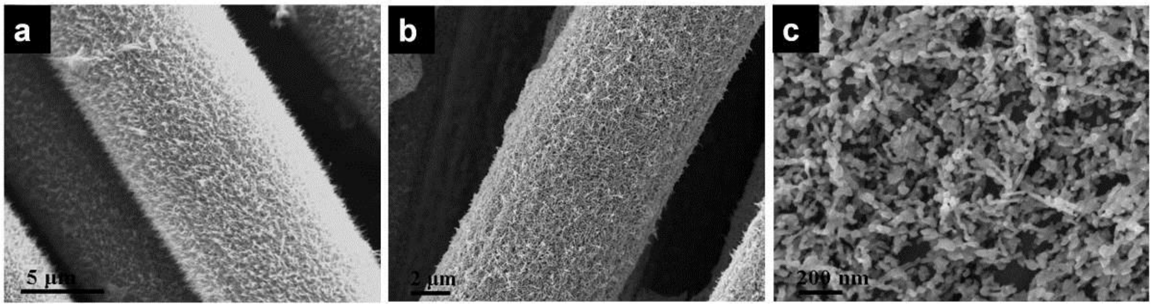

Figure S5. SEM images of (a) CC/V_2O_5, and (b, c) CC/p-VN.

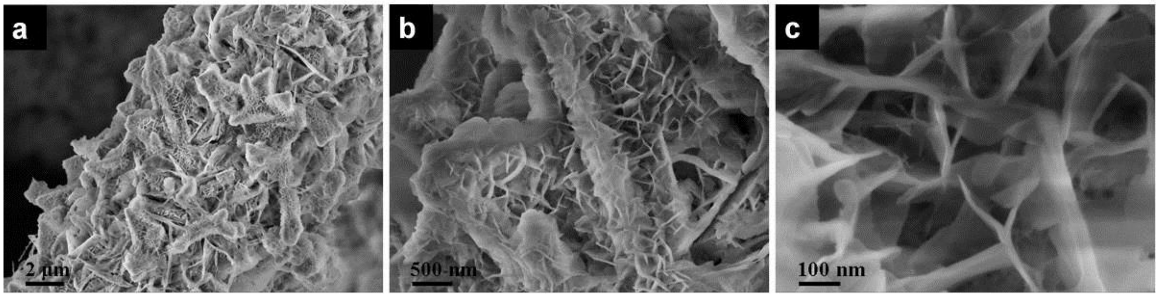

Figure S6. SEM images of CC/CW/Mn_3O_4.

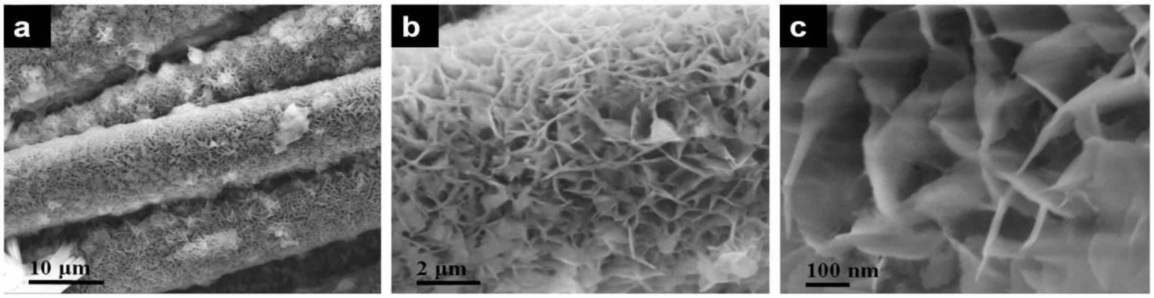

Figure S7. SEM images of CC/Mn_3O_4.

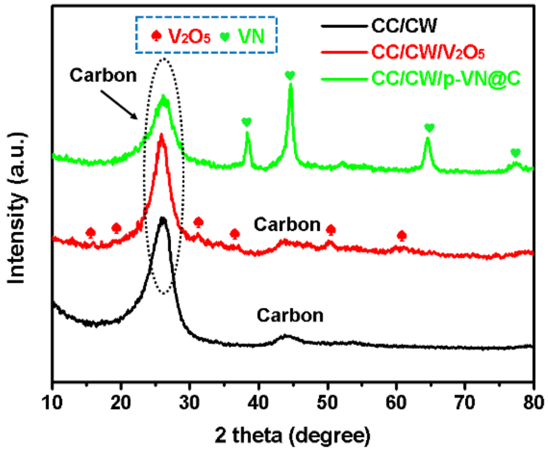

Figure S8. XRD patterns of CC/CW, CC/CW/V_2O_5, and CC/CW/p-VN@C.

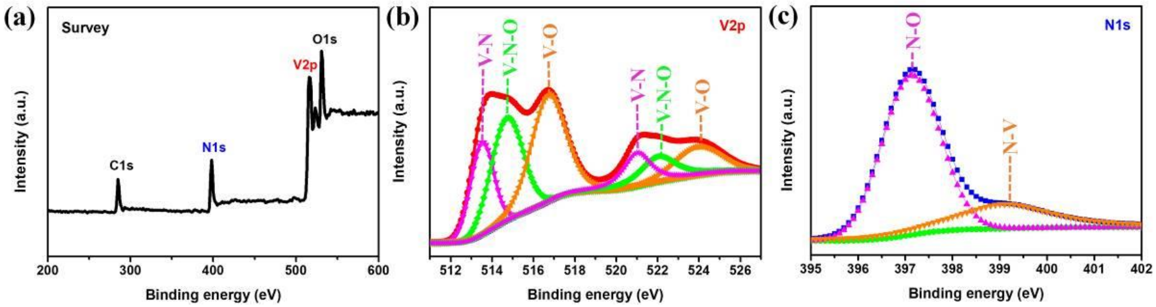

Figure S9. XPS spectra of CC/CW/p-VN@C. (a) survey, (b) V 2p, and (c) N 1s.

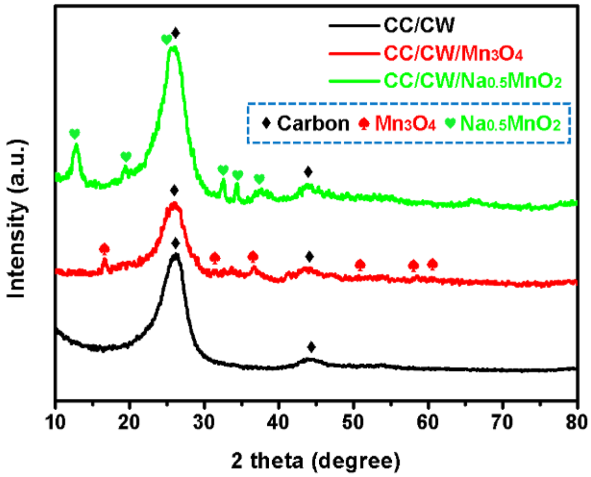

Figure S10. XRD patterns of CC/CW, CC/Mn_3O_4, and CC/CW/$Na_{0.5}MnO_2$.

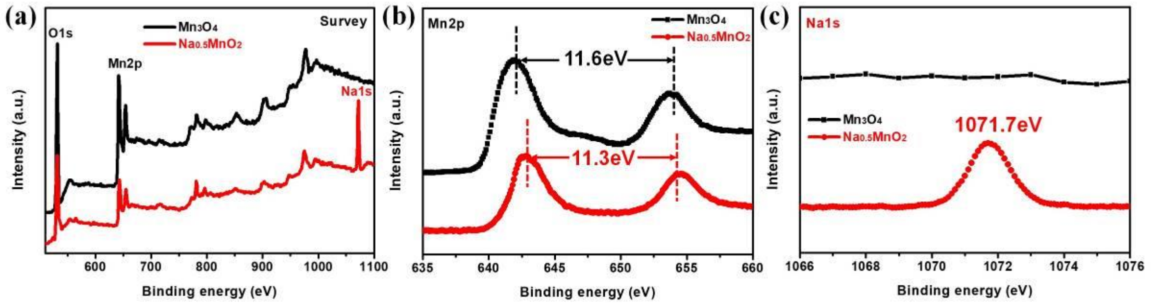

Figure S11. XPS spectra of Mn_3O_4 and $Na_{0.5}MnO_2$. (a) survey, (b) Mn 2p, and (c) Na 1s.

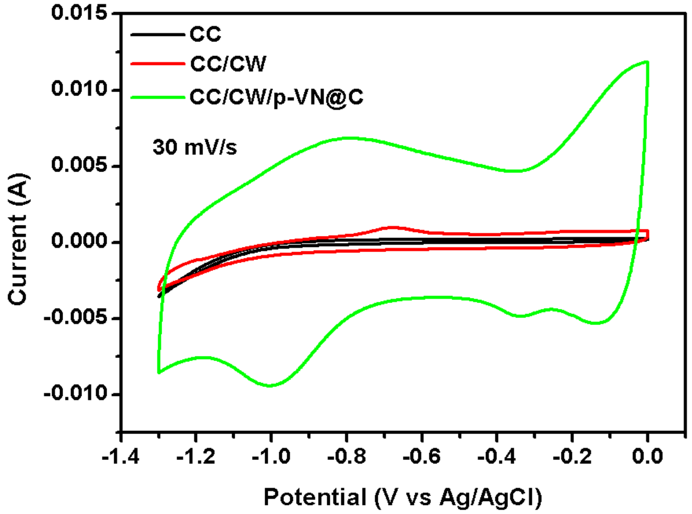

Figure S12. The CV curves of CC, CC/CW and CC/CW/p-VN@C electrodes at a scan rate of 30 mV s^{-1}.

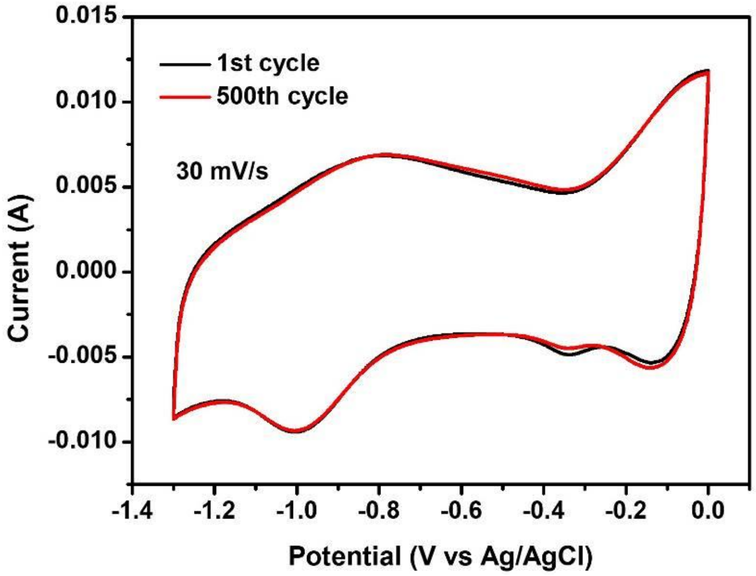

Figure S13. CV curves of CC/CW/p-VN@C electrode between -1.3 and 0 V (vs. Ag/AgCl) at first and 500th cycle.

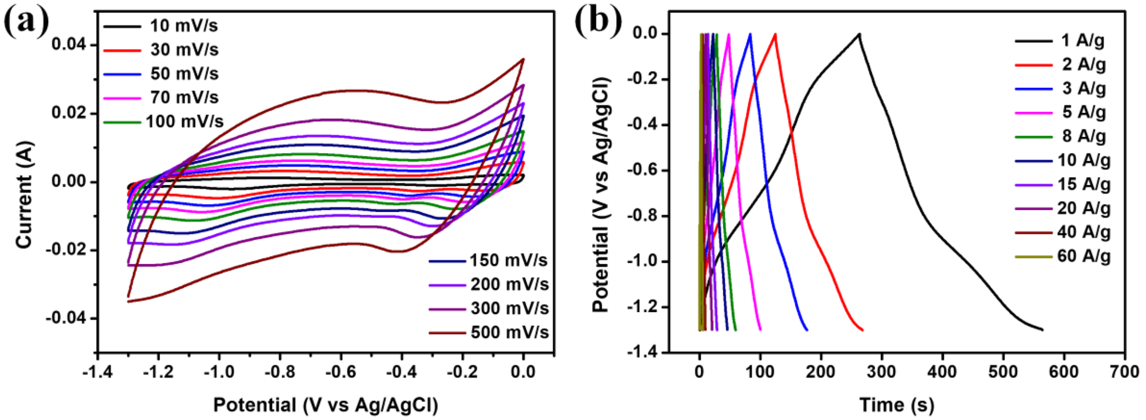

Figure S14. (a) CV curves of CC/p-VN electrode at different scan rates, (b) GCD curves of CC/p-VN electrode at different current densities.

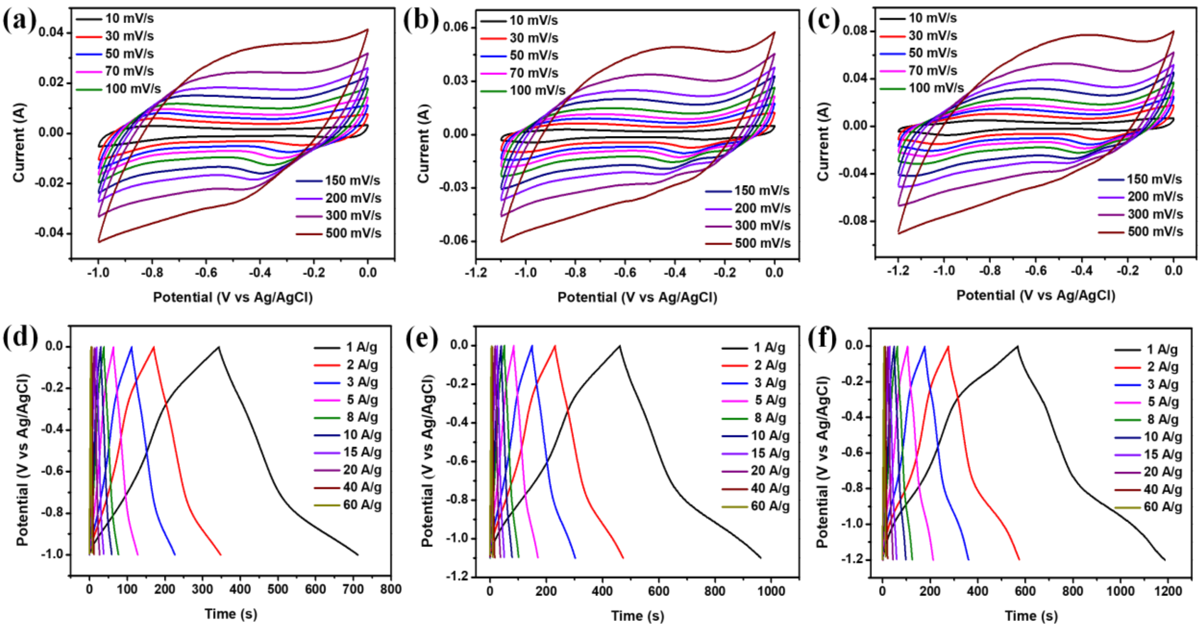

Figure S15. CV curves of the CC/CW/p-VN@C electrode in different potential windows of (a) -1.0-0 V, (b) -1.1-0 V, and (c) -1.2-0 V at different scan rates. GCD curves of the CC/CW/p-VN@C electrode in different potential windows of (d) -1.0-0 V, (e) -1.1-0 V, and (f) -1.2-0 V at different current densities.

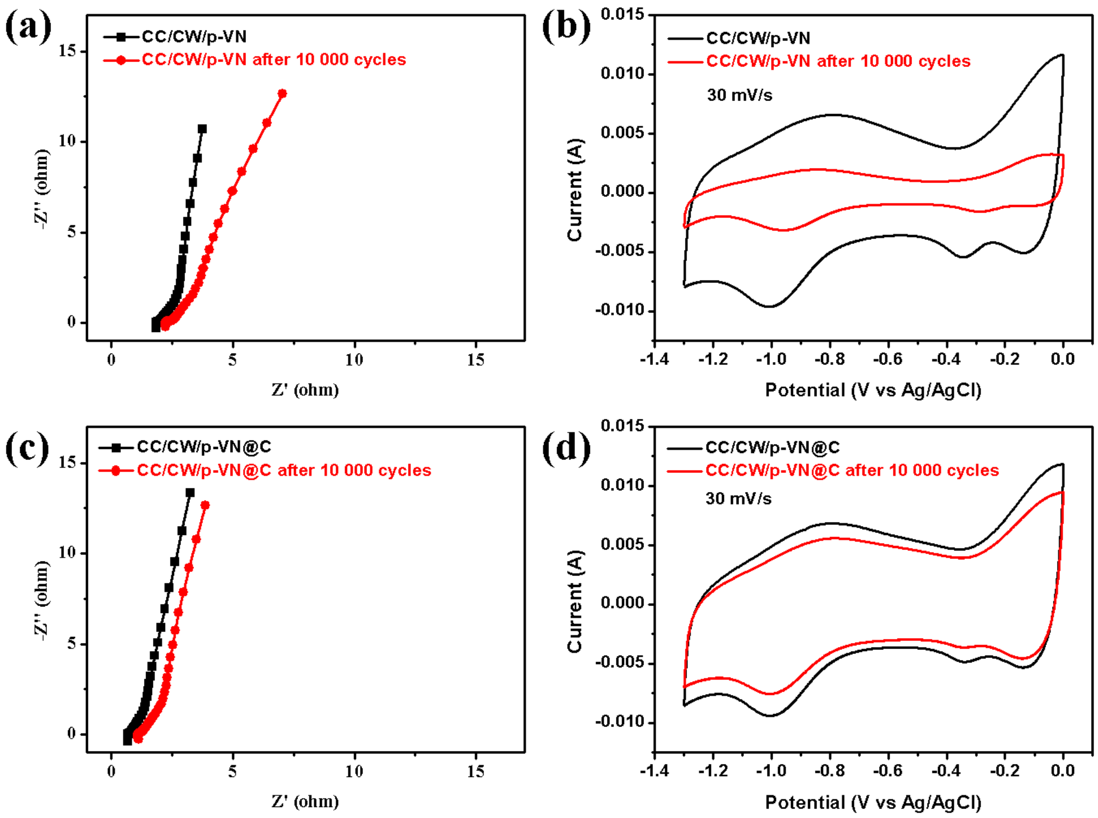

Figure S16. (a) EIS and (b) CV comparison of CC/CW/p-VN electrode before and after 10 000 cycles. (c) EIS and (d) CV comparison of CC/CW/p-VN@C electrode before and after 10 000 cycles.

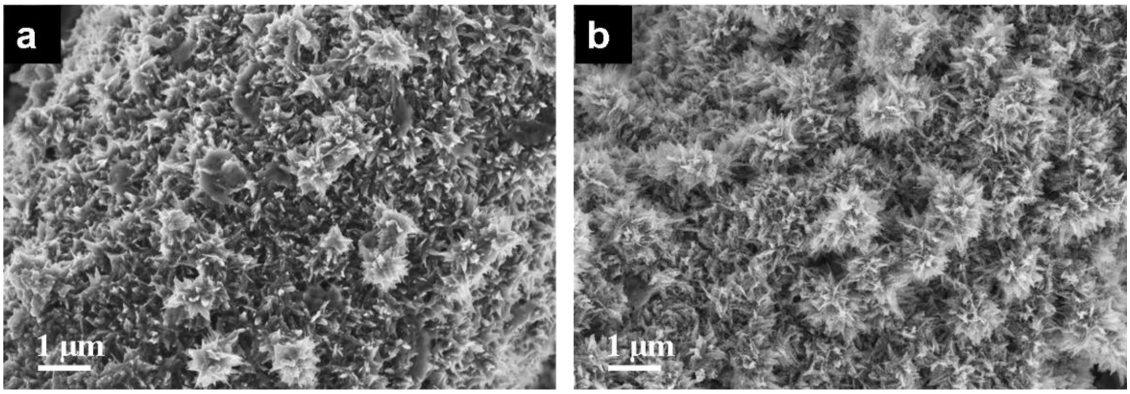

Figure S17. The SEM images of (a) CC/CW/p-VN and (b) CC/CW/p-VN@C electrodes after 10 000 cycles.

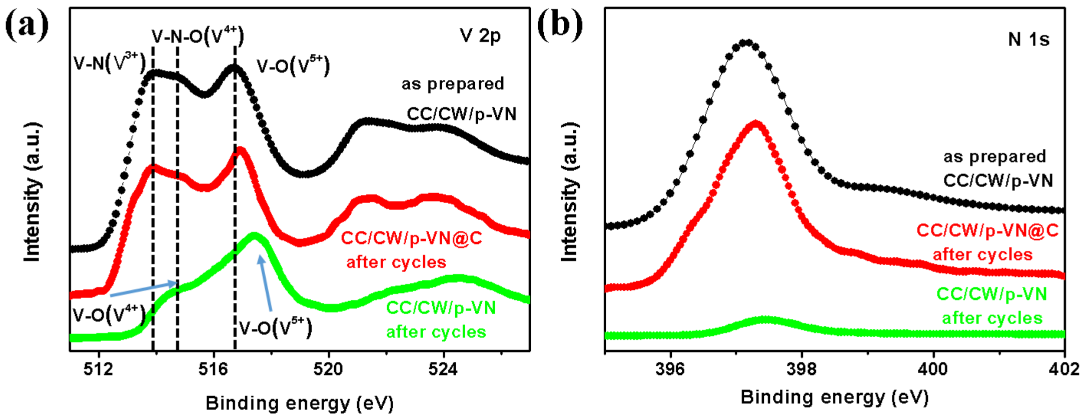

Figure S18. Core level (a) V 2p and (b) N 1s XPS spectra collected for CC/CW/p-VN and CC/CW/p-VN@C electrodes before and after testing for 10 000 cycles

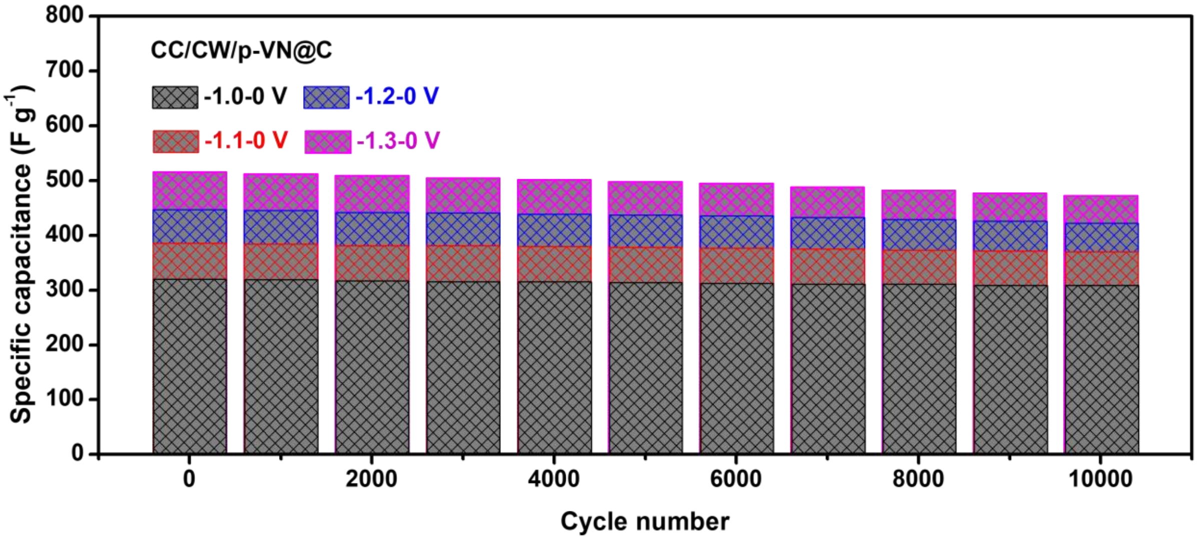

Figure S19. Cycle performance of CC/CW/p-VN@C electrode in different potential windows of -1.0–0, -1.1–0, -1.2–0, and -1.3–0 V at a current density of 5 A g^{-1}.

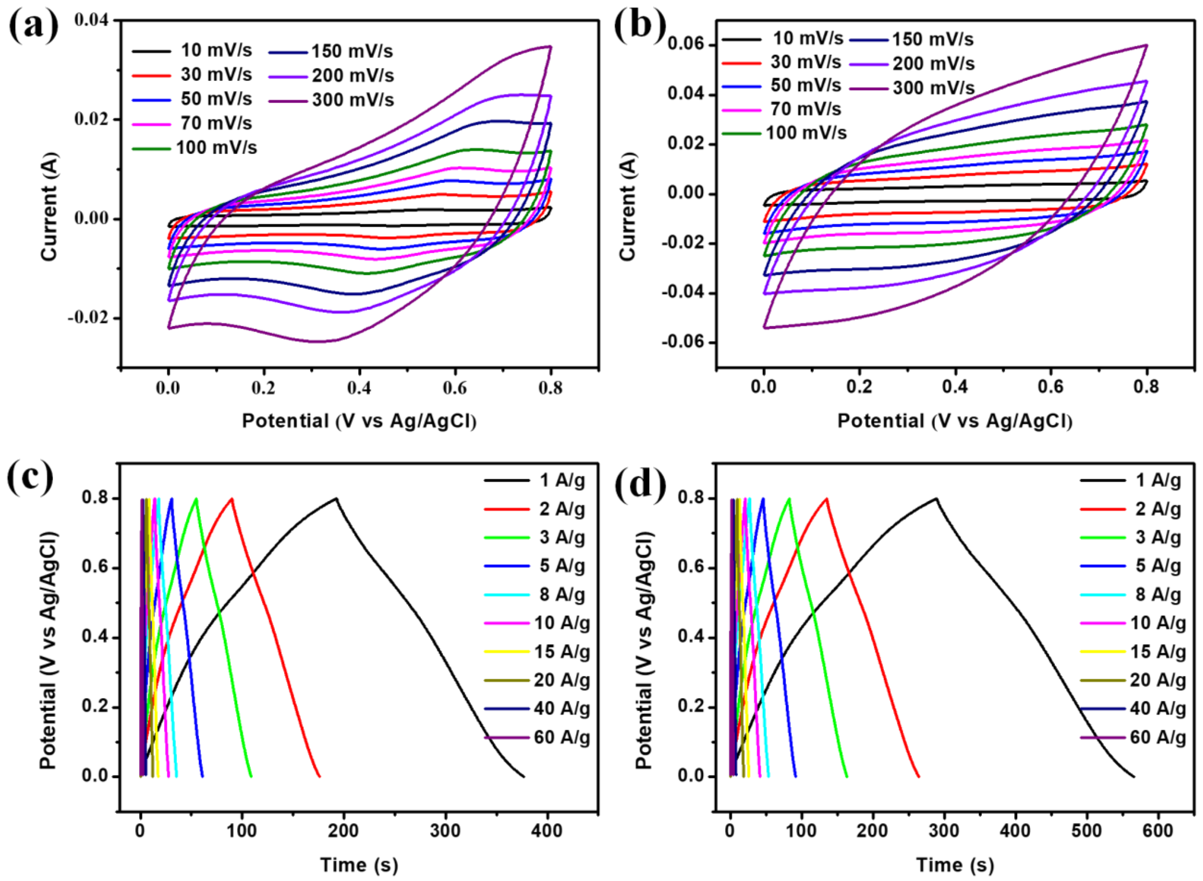

Figure S20. CV curves of (a) CC/Mn_3O_4 and (b) CC/CW/Mn_3O_4 in a potential windows of 0–0.8 V at different scan rates. GCD curves of (c) CC/Mn_3O_4 and (d) CC/CW/Mn_3O_4 in a potential window of 0–0.8 V at different current densities.

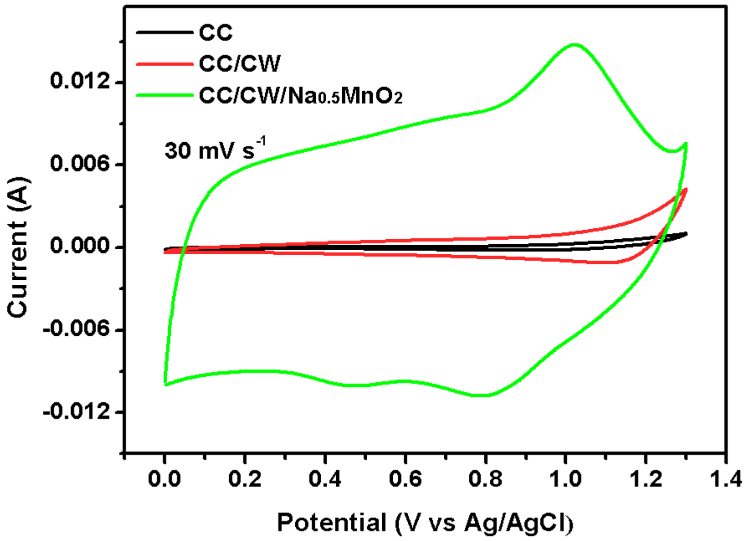

Figure S21. The CV curves of CC, CC/CW and CC/CW/$Na_{0.5}MnO_2$ electrodes at a scan rate of 30 mV s^{-1}.

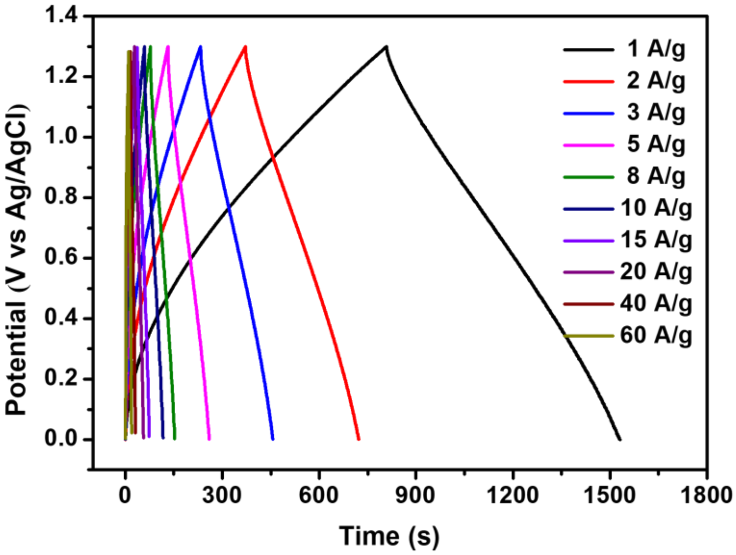

Figure S22. GCD curves of the CC/CW/$Na_{0.5}MnO_2$ electrode in a potential window of 0–1.3 V at different current densities.

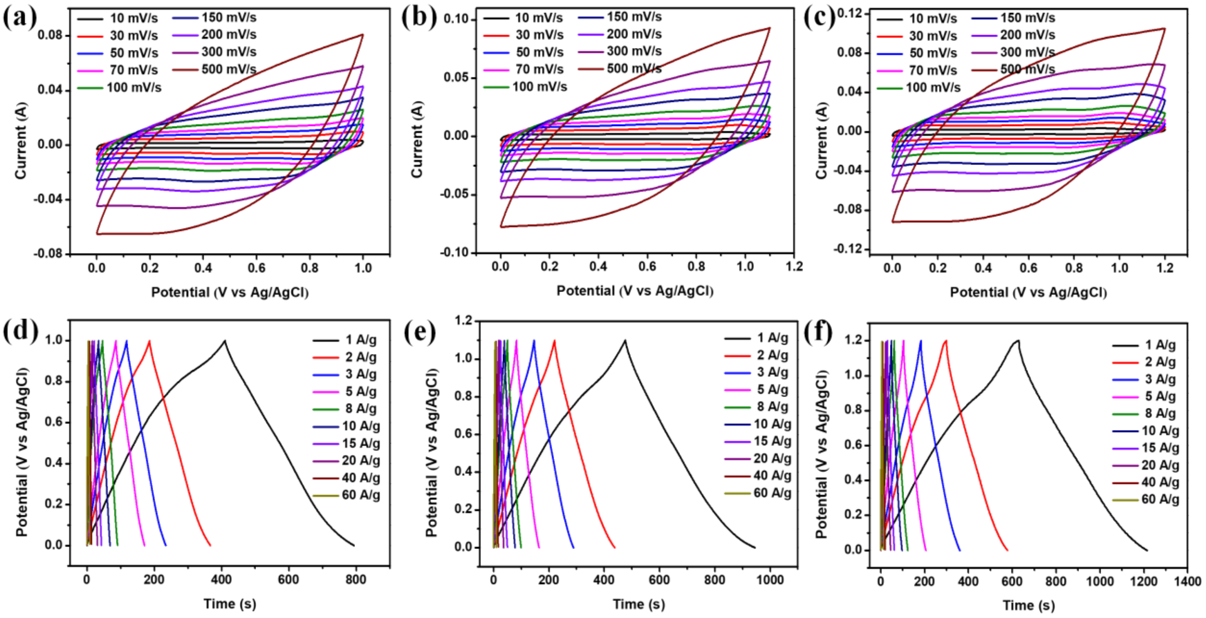

Figure S23. CV curves of the CC/CW/$Na_{0.5}MnO_2$ electrode in different potential windows of (a) 0–1.0 V, (b) 0–1.1 V, and (c) 0–1.2 V at different scan rates. GCD curves of the CC/CW/$Na_{0.5}MnO_2$ electrode in different potential windows of (d) 0–1.0 V, (e) 0–1.1 V, and (f) 0–1.2 V at different current densities.

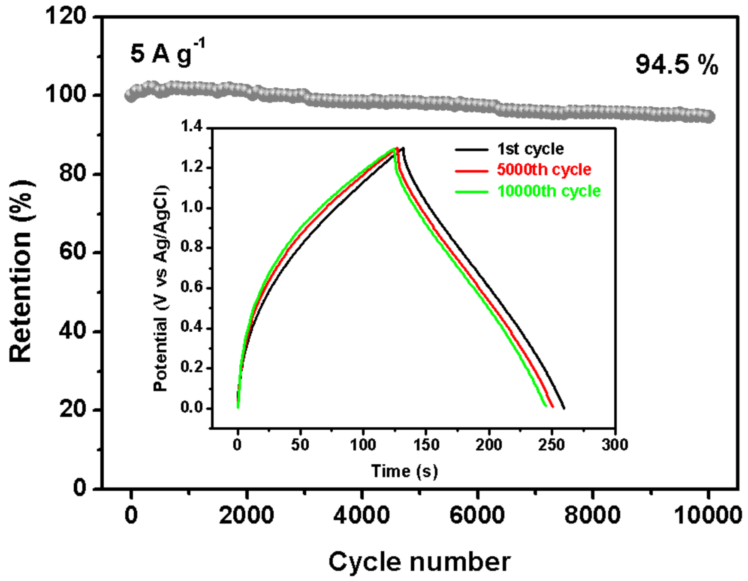

Figure S24. Long-term cycling performance of the CC/CW/$Na_{0.5}MnO_2$ cathode at a current density of 5 A g^{-1} for 10 000 cycles and the corresponding GCD curves of initial-in between-final cycles.

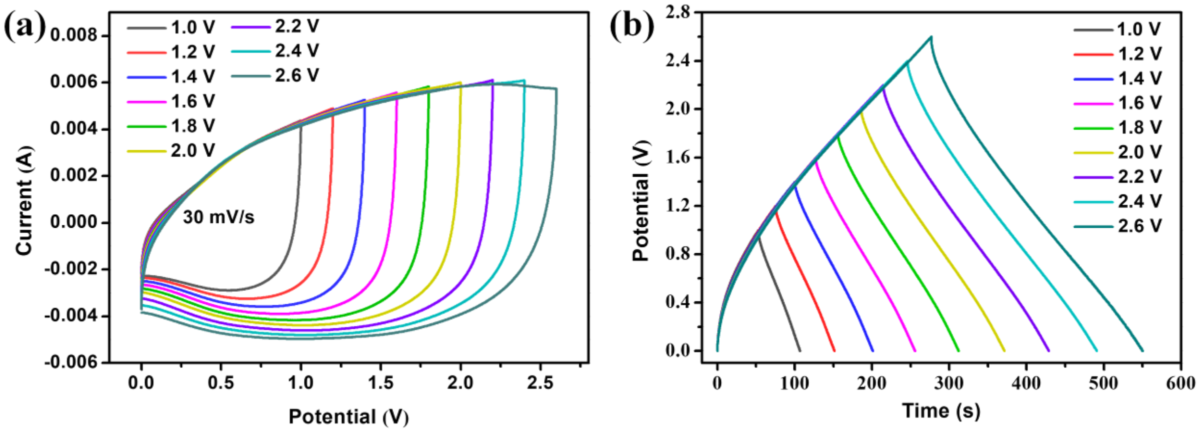

Figure S25. (a) CV curves of the CC/CW/p-VN@C//CC/CW/$Na_{0.5}MnO_2$ ASC in different voltage windows at a scan rate of 30 mV s^{-1}. (b) GCD curves of the CC/CW/p-VN@C//CC/CW/$Na_{0.5}MnO_2$ ASC in different voltage windows at a current density of 1 A g^{-1}.

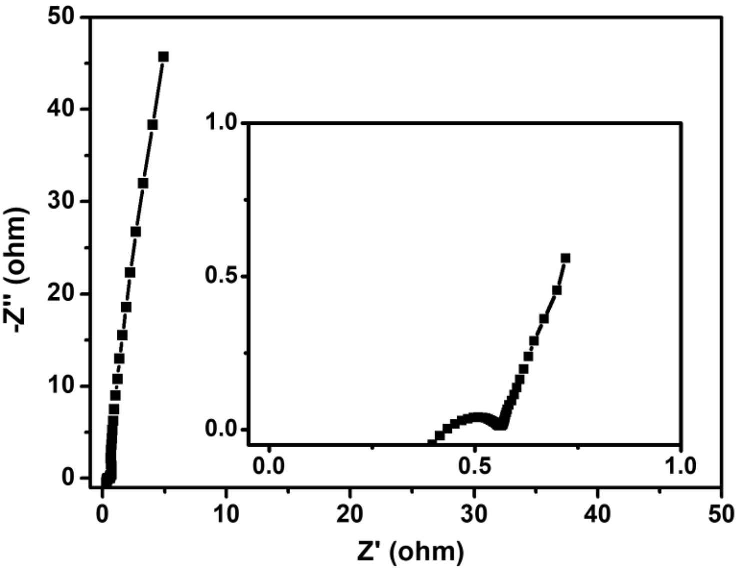

Figure S26. EIS spectrum of the CC/CW/p-VN@C//CC/CW/$Na_{0.5}MnO_2$ ASC.

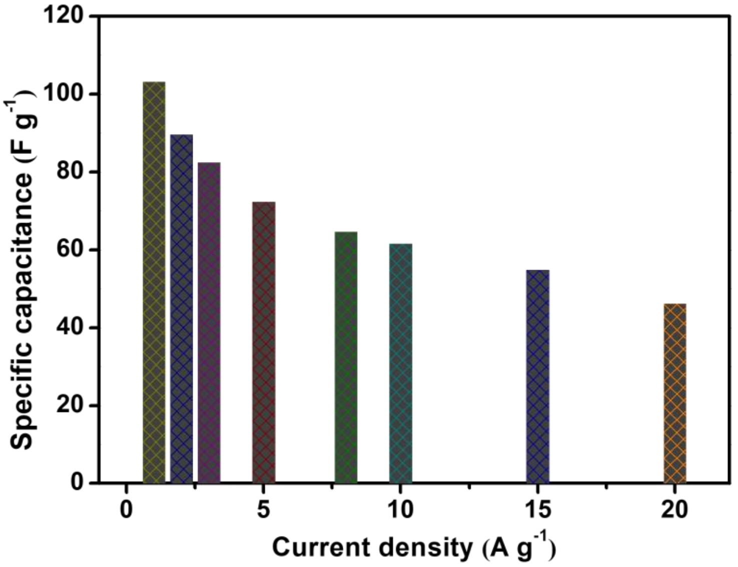

Figure S27. The specific capacitances of CC/CW/p-VN@C//CC/CW/$Na_{0.5}MnO_2$ ASC at different current densities.

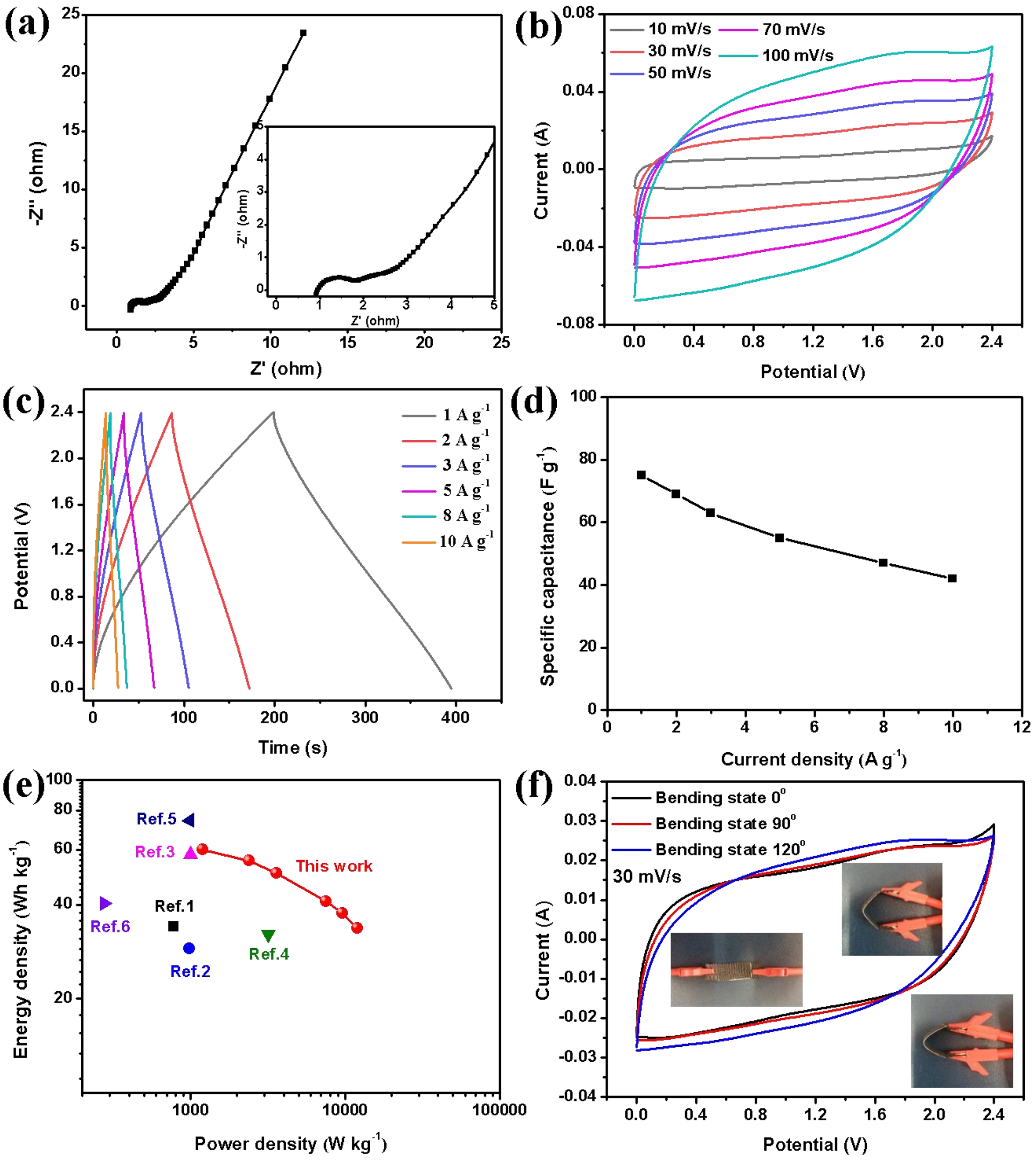

Figure S28. The electrochemical performance of all-solid-state CC/CW/p-VN@C//CC/CW/$Na_{0.5}MnO_2$ fexible ASC. (a) EIS spectrum, (b) CV curves at different scan rates, (c) GCD curves at different current densities, (d) specific capacitances at different current densities, (e) Ragone plots of values reported in literatures and this work, (f) CV curves of the flexible ASC under different bending conditions. The insets of (f) show the photographic images of the device under different bending states.

References

[1] X. Li, H. J. Wu, A. M. Elshahawy, L. Wang, S. J. Pennycook, C. Guan, J. Wang, *Adv. Funct. Mater.* **2018**, *28*, 1800036.

[2] X. M. Liu, W. J. Zang, C. Guan, L. Zhang, Y. H. Qian, A. M. Elshahawy, D. Zhao, S. J. Pennycook, J. Wang, *ACS Energy Lett.* **2018**, *3*, 2462.

[3] S. X. Sun, J. H. Luo, Y. Qian, Y. Jin, Y. Liu, Y. G. Qiu, X. Li, C. Fang, J. T. Han, Y. H. Huang, *Adv. Energy Mater.* **2018**, *8*, 1801080.

[4] Y. Li, J. Xu, T. Feng, Q. F. Yao, J. P. Xie, H. Xia, *Adv. Funct. Mater.* **2017**, 2*7*, 1606728.

[5] J. W. Choi, II. W. Ock, K. H. Kim, H. M. Jeong, J. K. Kang, *Adv. Funct. Mater.* **2018**, *28*, 1803695.

[6] S. J. Zhu, L. Li, J. B. Liu, H. T. Wang, T. Wang, Y. X. Zhang, L. L. Zhang, R. S. Ruoff, F. Dong, *ACS Nano* **2018**, *12*, 1033.
